# Isotretinoin Use and Liver Enzymes Changes: A Single-Center Study in Saudi Arabia

**DOI:** 10.7759/cureus.51263

**Published:** 2023-12-29

**Authors:** Mahdi Al Dhafiri, Feroze Kaliyadan, Sara Almukhaimar, Fatemah Alsultan, Elham Al Hayim, Roaa Alnaim, Alaa Aldossari

**Affiliations:** 1 Department of Dermatology, College of Medicine, King Faisal University, Al-Hofuf, SAU; 2 Department of Dermatology, Sree Narayana Institute of Medical Sciences, Ernakulam, IND; 3 Medical School, College of Medicine, King Faisal University, Al-Hofuf, SAU

**Keywords:** saudi arabia, acne vulgaris, alanine aminotransferase, aspartate aminotransferase, liver enzymes, isotretinoin

## Abstract

Introduction

Isotretinoin is a bioactive retinoic acid variant that is taken orally to treat moderate to severe acne vulgaris. One of the adverse effects of isotretinoin is elevated liver enzymes. This study estimated the prevalence of liver enzyme changes during isotretinoin use among dermatology clinic patients in Al-Ahsa, Kingdom of Saudi Arabia.

Methods

This study was a retrospective analysis that reviewed the medical data of 97 patients with acne at the King Faisal University Polyclinic who were taking systemic isotretinoin. It determined the baseline, second, and last readings of aspartate aminotransferase (AST) and alanine aminotransferase (ALT). Among the 97 patients, 67 (69.1%) were female and 30 (30.9%) were male.

Results

Of the patients, 41 (50.6%) weighed 51-70 kg, and 45 (46.4%) were 21-23 years old. The age of acne onset was 20 years or younger. Most patients had a starting isotretinoin dose of 10-20 mg and an ending dose of 30-40 mg over six months. Ninety (92.8%) patients had not used isotretinoin in the past. Before treatment, AST was elevated in three (3.1%) patients, and ALT was elevated in two (2.1%) patients. In the last readings, AST was elevated in eight (8.2%) patients, and ALT was elevated in four (4.1%) patients.

Conclusion

The result of this study indicates that the incidence of high levels of AST and ALT with oral isotretinoin was low. So frequent laboratory monitoring is not recommended since the elevation was not associated with any morbidity and carries financial and emotional burdens. An exception is patients with higher body weight, males, and those whose acne started at age 16-19, in whom frequent monitoring may be considered for AST more than ALT.

## Introduction

Isotretinoin is an active form of retinoic acid derived from vitamin A that predominantly affects the lipid composition of the skin by decreasing sebaceous gland size and sebum production [1].

There is a recent hypothesis that taking isotretinoin orally increases the expression of the transcription factor p53. Increasing the expression of p53 regulates many transcription factors implicated in the development of acne vulgaris, including FoxO1, androgen receptor, and key genes involved in autophagy and apoptosis induction [2]. Regarding the dosage, a recent systemic review showed that severe acne reacts better to standard (0.5 mg/kg/day) or higher fixed daily dosages of isotretinoin (1.6 mg/kg/day) [3].

Acne is a common inflammatory skin condition that affects the skin’s pilosebaceous units [4,5]. Isotretinoin is indicated for treating moderate to severe acne that is unresponsive to other therapies [2,6]. An adequate treatment course can induce remission by regulating cell division and differentiation and inhibiting sebum production [7].

Isotretinoin is linked to several side effects of which teratogenicity is the most harmful. However, dry, cracked lips, skin, and nasal mucosa are the most common mucocutaneous side effects [8,9]. Increased levels of liver enzymes including aspartate transaminase (AST) and alanine transaminase (ALT) and lipid changes including changes in triglyceride (TG) and total cholesterol (TC) levels are other adverse effects linked to systemic isotretinoin [10,11]. Local studies have identified elevated AST and ALT levels in laboratory monitoring during acne treatment [12,13].

Studies have reported conflicting findings on the prevalence of isotretinoin-associated liver enzyme changes. Because of the lack of consensus, we analyzed the prevalence of liver enzyme changes in patients who were prescribed isotretinoin at the King Faisal University (KFU) Polyclinic in Al-Ahsa, Saudi Arabia.

## Materials and methods

This study adopted a retrospective cohort design and collected patient data and laboratory test results from the KFU Polyclinic’s recording system. Before starting the study, ethical approval was obtained from the KFU Research Ethics Committee (approval number: KFU-REC-2023-SEP-ETHICS1107 dated September 6, 2023). The study included patients with acne vulgaris of all ages who received oral isotretinoin therapy along with baseline and follow-up lab testing to assess AST and ALT levels. Those who used isotretinoin for medical conditions other than acne and those who had only one or two readings were excluded from the study.

The following parameters were assessed: age, gender, weight, age of acne onset, age at which isotretinoin treatment began, dose of isotretinoin (starting dose and ending dose), duration of treatment, previous isotretinoin use, and liver enzymes lab test results.

Patients were regularly monitored; the laboratory findings of the AST and ALT levels were collected before the start of the treatment with isotretinoin (pre-treatment) and during the treatment. In this study, we considered three readings: the pre-treatment reading, one to three months after the start of the treatment, and four to six months after the start of the treatment (second and last readings). According to the analyzing reference laboratory, AST levels of 14-36 uIU/L were classified as normal and levels >36 uIU/L were classified as high. ALT levels of 9-52 U/L were classified as normal and levels >52 U/L were classified as high.

Data were entered into a Microsoft Excel document (Microsoft Corporation, Redmond, Washington, United States). Categorical variables are presented as numbers and percentages. Fischer Exact Test was used to identify the relationship between the patient's demographic and clinical characteristics with the normal and the abnormal liver enzyme levels. Statistical significance was defined as a p-value less than or equal to 0.05. The data were analyzed using IBM SPSS Statistics for Windows, Version 26.0 (Released 2019; IBM Corp., Armonk, New York, United States).

## Results

A total of 97 patients with liver enzyme data were included in the study. Among them, 45 (46.4%) were between the ages of 21 and 23 years, and most patients were female (n=67; 69.1%). Approximately half (n=42; 50.6%) of the patients weighed 51-70 kg. Nearly half of the patients (n=48; 49.5%) began isotretinoin treatment at age 20 or younger, and the most common age of acne onset was 19 years or older (n=47; 48.5%). The starting dose was typically 10-20 mg in 85 (87.6%) patients, whereas the ending dose was typically 30-40 mg in 49 (50.5%) patients. Most patients (n=52; 53.6%) had a treatment duration of six months, and seven (7.2%) patients had used isotretinoin previously (Table 1).

**Table 1:** Demographic and clinical characteristics of the patients (N = 97)

Study variables	n (%)
Age group	
≤20 years	21 (21.6%)
21–23 years	45 (46.4%)
>23 years	31 (32.0%)
Gender	
Male	30 (30.9%)
Female	67 (69.1%)
Weight (n = 83)	
30–50 kg	17 (20.5%)
51–70 kg	42 (50.6%)
>70 kg	24 (28.9%)
Age of acne onset	
11–15 years	19 (19.6%)
16–19 years	31 (32.0%)
>19 years	47 (48.5%)
Age at which isotretinoin treatment began	
≤20 years	48 (49.5%)
21–23 years	35 (36.1%)
>23 years	14 (14.4%)
Starting dose	
10–20 mg	85 (87.6%)
30–40 mg	12 (12.4%)
Ending dose	
10–20 mg	43 (44.3%)
30–40 mg	49 (50.5%)
>40 mg	5 (5.2%)
Duration of treatment	
<5 months	52 (53.6.%)
≥5 months	45 (46.4%)
Previous isotretinoin use	
Yes	7 (7.2%)
No	90 (92.8%)

Pre-treatment AST and ALT levels were normal in 94 (96.9%) and 95 (97.9%) patients, respectively. The second AST and ALT readings (during the first to the third month) were normal in 90 (92.8%) and 92 (94.8%) patients, respectively, whereas the last readings (during the fourth to the sixth month) were normal in 89 (91.8%) and 93 (95.9%) patients, respectively (Table 2).

**Table 2:** Liver enzyme parameters (N = 97) AST: aspartate aminotransferase; ALT: alanine aminotransferase AST values: normal 14–36 uIU/L, high >36 uIU/L; ALT values: normal 9–52 U/L, high >52 U/L

Parameters	n (%)
AST before treatment	
Normal	94 (96.9%)
High	3 (3.1%)
ALT before treatment	
Normal	95 (97.9%)
High	2 (2.1%)
Second AST reading (first to the third month)	
Normal	90 (92.8%)
High	7 (7.2%)
Second ALT reading (first to the third month)	
Normal	92 (94.8%)
High	5 (5.2%)
Last AST reading (fourth to the sixth month)	
Normal	89 (91.8%)
High	8 (8.2%)
Last ALT reading (fourth to the sixth month)	
Normal	93 (95.9%)
High	4 (4.1%)

Elevated pre-treatment AST levels were significantly associated with male gender (p = 0.028), an age of acne onset of 16-19 years (p = 0.037), and a weight of >70 kg (p = 0.029). By contrast, no significant relationships were observed between the last AST readings and any of the demographic and clinical variables (age group, gender, age of acne onset, age at which isotretinoin treatment began, starting dose, ending dose, duration of treatment, previous isotretinoin use, and weight group; all p > 0.05) (Table 3). 

**Table 3:** Relationship between first and last AST readings and the demographic and clinical characteristics of the patients (N = 97) AST: aspartate aminotransferase p-values were calculated using the Fisher exact test. ** Significant at p < 0.05 level

Factor	Pre-treatment AST readings		p-value	Last AST readings		p-value
	Normal N (%) (n = 94)	High N (%) (n = 3)		Normal N (%) (n = 89)	High N (%) (n = 8)	
Age group						
≤22 years	48 (51.1%)	3 (100%)	0.244	47 (52.8%)	4 (50.0%)	1.000
>22 years	46 (48.9%)	0 (0%)		42 (47.2%)	4 (50.0%)	
Gender						
Male	27 (28.7%)	3 (100%)	0.028 **	26 (29.2%)	4 (50.0%)	0.248
Female	67 (71.3%)	0 (0%)		63 (70.8%)	4 (50.0%)	
Age of acne onset						
11–15 years	19 (20.2%)	0 (0%)	0.037 **	15 (16.9%)	4 (50.0%)	0.111
16–19 years	28 (29.8%)	3 (100%)		30 (33.7%)	1 (12.5%)	
>19 years	47 (50.0%)	0 (0%)		44 (49.4%)	3 (37.5%)	
Age at which isotretinoin treatment began						
≤20 years	46 (48.9%)	2 (66.7%)	1.000	44 (49.4%)	4 (50.0%)	1.000
21–23 years	34 (36.2%)	1 (33.3%)		32 (36.0%)	3 (37.5%)	
>23 years	14 (14.9%)	0 (0%)		13 (14.6%)	1 (12.5%)	
Starting dose						
10–20 mg	82 (87.2%)	3 (100%)	1.000	77 (86.5%)	8 (100%)	0.590
30–40 mg	12 (12.8%)	0 (0%)		12 (13.5%)	0 (0%)	
Ending dose						
10–20 mg	40 (42.6%)	3 (100%)	0.232	37 (41.6%)	6 (75.0%)	0.217
30–40 mg	49 (52.1%)	0 (0%)		47 (52.8%)	2 (25.0%)	
>40 mg	5 (5.3%)	0 (0%)		5 (5.6%)	0 (0%)	
Duration of treatment						
<5 months	59 (62.8%)	1 (33.3%)	0.556	55 (61.8%)	5 (62.5%)	1.000
≥5 months	35 (37.2%)	2 (66.7%)		34 (38.2%)	03 (37.5%)	
Previous isotretinoin use						
Yes	6 (6.4%)	1 (33.3%)	0.203	6 (6.7%)	1 (12.5%)	0.464
No	88 (93.6%)	2 (66.7%)		83 (93.3%)	7 (87.5%)	
Weight						
30 – 50 kg	17 (21.3%)	0 (0%)	0.029 **	16 (21.1%)	1 (14.3%)	0.587
51 – 70 kg	42 (52.5%)	0 (0%)		37 (48.7%)	5 (71.4%)	
>70 kg	21 (26.3%)	3 (100%)		23 (30.3%)	1 (14.3%)	

Furthermore, we did not observe significant relationships between pre-treatment ALT levels and any of the demographic or clinical characteristics (all p > 0.05) except for a relationship between male gender and high ALT levels in the last readings (p = 0.008) (Table 4). 

**Table 4:** Relationship between the first and last ALT readings and the demographic and clinical characteristics of the patients (N = 88) ALT: alanine aminotransferase p-values were calculated using the Fisher exact test ** Significant at p < 0.05 level

Factor	Pre-treatment ALT readings		p-value	Last ALT readings		p-value
	Normal, n (%) (n = 95)	High, n (%) (n = 02)		Normal. n (%) (n = 92)	High, n (%) (n = 4)	
Age group						
≤22 years	49 (51.6%)	2 (100%)	0.496	47 (50.5%)	4 (100%)	0.119
>22 years	46 (48.4%)	0 (0%)		46 (49.5%)	0 (0%)	
Gender						
Male	28 (29.5%)	2 (100%)	0.093	26 (28.0%)	4 (100%)	0.008 **
Female	67 (70.5%)	0 (0%)		67 (72.0%)	0 (0%)	
Age of acne onset						
11–15 years	19 (20.0%)	0 (0%)	1.000	18 (19.4%)	1 (25.0%)	0.526
16–19 years	30 (31.6%)	1 (50.0%)		29 (31.2%)	2 (50.0%)	
>19 years	46 (48.4%)	1 (50.0%)		46 (49.5%)	1 (25.0%)	
Age at which isotretinoin treatment began						
≤20 years	48 (50.5%)	0 (0%)	0.253	46 (49.5%)	2 (50.0%)	1.000
21–23 years	33 (34.7%)	2 (100%)		33 (35.5%)	2 (50.0%)	
>23 years	14 (14.7%)	0 (0%)		14 (15.1%)	0 (0%)	
Starting dose						
10–20 mg	83 (87.4%)	2 (100%)	1.000	81 (87.1%)	4 (100%)	1.000
30–40 mg	12 (12.6%)	0 (0%)		12 (12.9%)	0 (0%)	
Ending dose						
10–20 mg	41 (43.2%)	2 (100%)	0.295	40 (43.0%)	3 (75.0%)	0.465
30–40 mg	49 (51.6%)	0 (0%)		48 (51.6%)	1 (25.0%)	
>40 mg	5 (5.3%)	0 (0%)		5 (5.4%)	0 (0%)	
Duration of treatment						
<5 months	58 (61.1%)	2 (100%)	0.523	57 (61.3%)	3 (75.0%)	1.000
≥5 months	37 (38.9%)	0 (0%)		36 (38.7%)	1 (25.0%)	
Previous isotretinoin use						
Yes	7 (7.4%)	0 (0%)	1.000	6 (6.5%)	1 (25.0%)	0.263
No	88 (92.6%)	2 (100%)		87 (93.5%)	3 (75.0%)	
Weight						
30–50 kg	17 (20.7%)	0 (0%)	0.494	16 (20.0%)	1 (33.3%)	0.116
51–70 kg	42 (51.2%)	0 (0%)		42 (52.5%)	0 (0%)	
>70 kg	23 (28.0%)	1 (100%)		22 (27.5%)	2 (66.7%)	

## Discussion

Previous studies have identified changes in liver enzyme (AST and ALT) levels as a side effect of oral isotretinoin treatment [10,12]. Some studies have reported a mild to moderate increase in liver enzymes as the most common side effect [14], whereas others have reported no elevation in liver enzymes [15].

Our study investigated the prevalence of liver enzyme changes during isotretinoin use in patients with acne. In pre-treatment, second, and last readings, the prevalence rates of AST elevation were 3 (3.1%), 7 (7.2%), and 8 (8.2%), respectively, and the rates of elevated ALT levels were 2 (2.1%), 5 (5.2%), and 4 (4.1%), respectively. A recent retrospective study of 143 individuals with moderate or severe acne vulgaris who used oral isotretinoin reported similar findings: AST levels were higher in the second and last readings compared with the baseline, whereas ALT levels did not change significantly [16]. In another recent study, including 200 patients, 20 (10%) and 15 (7.5%) patients exhibited elevated AST and ALT levels at baseline, respectively, followed by 30 (15%) and 40 (20%) patients in the third month and 91 (45.5%) and 20 (10%) patients in the sixth month [17].

A study conducted by Alajaji et al. in Al Qassim, Saudi Arabia, which included 407 patients, reported increased AST and ALT levels in 21 (5.4%) and 48 (12.3%) patients at baseline, 12 (5.4%) and 25 (11.4%) patients during follow-up, and 15 (3.9%) and 34 (9.0%) patients in the last lab test. However, compared with the baseline, the liver enzyme levels did not increase significantly [12].

Among 386 patients included in a study in Riyadh, Saudi Arabia, the prevalence rates of high AST were 8 (2.2%), 7 (1.9%), and 14 (3.8%) at baseline, the first follow-up, and the second follow-up, respectively. The prevalence rates of high ALT levels were 47 (12.7%), 49 (13.2%), and 37 (10.0%), respectively, although the changes were not statistically significant [13].

In the present study, male gender was associated with elevated pre-treatment AST levels and last ALT readings. Similarly, another study reported a higher prevalence of liver abnormalities in males than in females [14]. However, according to a different study, gender was not significantly associated with AST or ALT levels [16].

Our results showed no significant association between the dose and duration of treatment and AST and ALT levels; earlier studies have reached similar conclusions, demonstrating that dose and duration of treatment were not significantly associated with increased liver enzyme levels [8,12,14]. In contrast to this, Abd-Elaziz et al. indicated that the dose of isotretinoin and duration of treatment were significantly associated with changes in AST and ALT levels. Patients who took 80 mg/day and used oral isotretinoin for four to six months were more likely to have increased liver enzymes [18].

Strengths and limitations

The findings of this research provide insights into the relationships of demographic and clinical variables, specifically age, gender, weight, dosage, duration of treatment, and previous isotretinoin use, with liver enzyme levels that have not been included in previous studies.

A limitation of this study was that BMI could not be assessed because of the lack of height data. Furthermore, this was a single-center study with a small sample size.

## Conclusions

The findings of this study indicate that oral isotretinoin can cause an elevation in ALT and AST levels but the incidence of these laboratory abnormalities is low, and the elevation was not associated with significant morbidity. We also found that patients with higher body weight, male gender, and those whose acne started at age 16-19 are at higher risk of laboratory abnormalities and may require more frequent laboratory monitoring. Our findings support less frequent laboratory monitoring for acne patients on isotretinoin who had normal baseline labs. Frequent laboratory monitoring of these patients carries financial and emotional implications and lacks strong evidence to support this practice.
